# Synergistic antitumor efficacy against the EGFRvIII^+^HER2^+^ breast cancers by combining trastuzumab with anti-EGFRvIII antibody CH12

**DOI:** 10.18632/oncotarget.6111

**Published:** 2015-10-14

**Authors:** Wen Xu, Yanyu Bi, Jiqin Zhang, Juan Kong, Hua Jiang, Mi Tian, Kesang Li, Biao Wang, Cheng Chen, Fei Song, Xiaorong Pan, Bizhi Shi, Xianming Kong, Jianren Gu, Xiumei Cai, Zonghai Li

**Affiliations:** ^1^ Medical School of Fudan University, Shanghai, China; ^2^ State Key Laboratory of Oncogenes & Related Genes, Shanghai Cancer Institute, Renji Hospital, Shanghai Jiaotong University School of Medicine, Shanghai, China

**Keywords:** EGFRvIII^+^HER2^+^ breast cancers, EGFRvIII, CH12, trastuzumab resistance, STAT3

## Abstract

Although Trastuzumab, an anti-HER2 antibody, benefits certain patients with HER2-overexpressing breast cancer, *de novo* or acquired trastuzumab resistance remains a haunting issue. EGFRvIII, co-expressing with HER2 in some breast tumors, indicates a poor clinical prognosis. However, the role of EGFRvIII in the function of trastuzumab is not clear. Here, we demonstrated that EGFRvIII overexpression contributed to *de novo* trastuzumab resistance and the feedback activation of STAT3 caused by trastuzumab also resulted in acquired resistance in EGFRvIII^+^HER2^+^ breast cancers. CH12, a highly effective anti-EGFRvIII monoclonal antibody that preferentially binds to EGFRvIII, significantly suppressed the growth of EGFRvIII^+^HER2^+^ breast cancer cells *in vitro* and *in vivo*. Importantly, CH12 in combination with trastuzumab had a synergistic inhibitory effect on EGFRvIII^+^HER2^+^ breast cancers *in vitro* and *in vivo* via attenuating the phosphorylation of EGFR and HER2 and their downstream signal pathways more effectively and reversing STAT3 feedback activation. Moreover, the combination therapy suppressed angiogenesis and induced cell apoptosis significantly. Together, these results suggested a synergistic efficacy of the combination of trastuzumab with CH12 against EGFRvIII^+^HER2^+^ breast cancers, which might be a potential clinical application in the future.

## INTRODUCTION

Human epidermal growth factor receptor 2 (HER2), a receptor tyrosine kinase (RTK) that regulates cell growth and differentiation signaling pathways, is highly overexpressed in approximately 20% to 25% of breast cancers, leading to an aggressive tumor phenotype and a dismal prognosis [1, 2]. Trastuzumab, a humanized antibody targeting HER2, shows considerable clinical efficacy and extends the overall survival of certain patients with HER2-overexpressing breast cancer [3–5]. However, the overall response rate to trastuzumab-containing therapies remains modest: ~26% when used as a single therapy and 40-60% when used in combination with systemic chemotherapy [5–8]. Many patients do not respond to initial trastuzumab treatment (*de novo* resistance), and many trastuzumab-responsive patients develop resistance after continuous treatment (acquired resistance) [9, 10]. Two major categories of trastuzumab resistance mechanisms have been proposed: (I) *De novo* resistance due to genetic alterations of receptor tyrosine kinases (RTKs) and their downstream signaling targets, such as aberrant activation of the PI3K/AKT pathway due to phosphatase and tensin homolog (PTEN) deficiency or PIK3CA gene activating mutations [11, 12], and the accumulation of truncated HER2 receptors (p95HER2) that lack the trastuzumab-binding domain [13]; and (II) acquired resistance primarily due to the acquisition of alternative RTK or feedback signal activation that compensate for HER2 inhibition after trastuzumab treatment [14–16].

EGFR, an essential RTK, playing an vital role in cell differentiation, proliferation, and survival in a number of human cancers, also contribute to both *de novo* and acquired trastuzumab resistance [14, 17]. Accumulating reports have demonstrated that EGFRvIII, the most common EGFR mutant forms with constitutively activated kinase domain [18, 19], expresses in various human cancers, including breast cancer, and it has not been detected in normal adult human tissue [20, 21]. EGFRvIII expression was detected in approximately 5% of primary breast cancer cases and contributes to cancer stem cell phenotypes in invasive breast carcinomas [21]. Furthermore, approximately 40% of HER2-positive primary breast tumors were found to co-express EGFRvIII, and, even more striking, 75% of HER2-positive metastatic lymph node specimens co-expressed EGFRvIII [22].

EGFRvIII is posited to be involved in tumorigenicity, invasiveness, and metastasis in breast cancers [22–24]. Several strategies against EGFRvIII-positive tumors have been explored. For instance, anti-EGFRvIII antibodies, such as mAb 806 and CH12, which selectively bind to a cancer-specific epitope of EGFR or EGFRvIII, have been demonstrated to be capable of efficiently inhibiting the growth of EGFRvIII-positive tumor xenografts [21, 25, 26]. However, it needs to be determined whether these antibodies have efficacy against breast tumors with EGFRvIII overexpression.

Considering the co-expression of HER2 and EGFRvIII in breast cancers, we predicted that HER2 and EGFRvIII might cooperate for tumor growth, and EGFRvIII expression might contribute to trastuzumab resistance. However, to date, no treatment strategies have been explored on EGFRvIII^+^HER2^+^ breast cancers. Therefore, in this study, we examined the combination effect of trastuzumab and CH12 on the EGFRvIII^+^HER2^+^ breast cancer cells and the molecular mechanisms underlying their antitumor effects.

## RESULTS

### EGFRvIII overexpression decreased the sensitivity of breast cancers to trastuzumab

**Figure 1:**
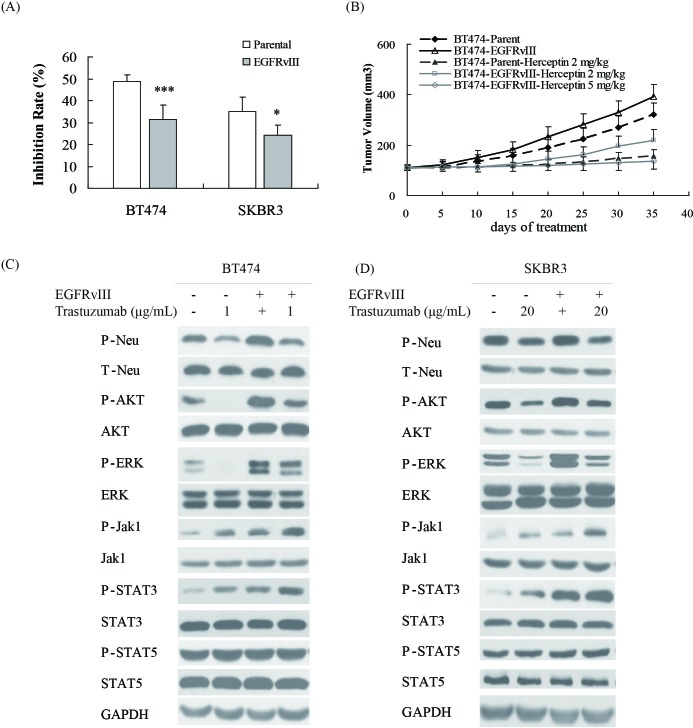
EGFRvIII overexpression decreased the sensitivity of breast cancers to trastuzumab **A.** Cell proliferation of parental BT474 and SKBR3 cell lines and their corresponding EGFRvIII overexpression sublines upon treatment with trastuzumab at a concentration of 1 μg/mL for BT474 and at a concentration of 20 μg/mL for SKBR3. Data are expressed as the inhibition rate of cell growth in triplicate experiments (Bars, SD). Statistical signiﬁcance is indicated *versus* parental cells *P < 0.05, **P < 0.01, ***P < 0.001. **B.** Growth curve of xenografts derived from either BT474 parental or EGFRvIII overexpression subline upon treatment with vehicle or trastuzumab at a concentration of 2 and 5 mg/kg intraperitoneally, once a week for 2 weeks. The data are expressed as mean tumor volumes ± SE. **C.**, **D.** Changes of EGFR or HER2 relevant signaling after trastuzumab treament in BT474 and BT474-EGFRvIII **C.** or SKBR3 and SKBR3-EGFRvIII **D.** cells.

The EGFRvIII encoding sequence was introduced into the HER2-positive breast cancer cell lines BT474 and SKBR3, and the established EGFRvIII^+^HER2^+^ cells (Figure S1) were less sensitive to trastuzumab than their parental cells *in vitro* (Figure 1A). Furthermore, the antitumor efficacy of trastuzumab in BT474-EGFRvIII xenografts in nude mice was slightly weaker than that in parental BT474 model (Figure 1B). The inhibition rate of trastuzumab at a concentration of 2 mg/kg was 51% in BT474-parental xenograft, while it was 43.7% in BT474-EGFRvIII model (*P* < 0.01).

To determine the molecular mechanism underlying EGFRvIII-mediated trastuzumab resistance, the downstream signaling of EGFR was analyzed. In EGFRvIII^+^HER2^+^ cell lines, ERK, AKT, Jak1 and STAT3, were activated compared with EGFRvIII^−^HER2^+^ cell lines (Figure 1C and 1D). After trastuzumab treatment, both AKT and ERK phosphorylation were less downregulated while Jak1 and STAT3 phosphorylation were unregulated more obviously in EGFRvIII^+^HER2^+^ than in EGFRvIII^−^HER2^+^ cancer cell lines (Figure 1C and 1D). Together, EGFRvIII overexpression might decrease the sensitivity of breast cancer cells to trastuzumab *via* constitutively activating EGFR downstream signals including ERK, AKT, and Jak1/STAT3.

### STAT3 activation contributed to acquired trastuzumab resistance in EGFRvIII^+^HER2^+^ breast cancer

**Figure 2:**
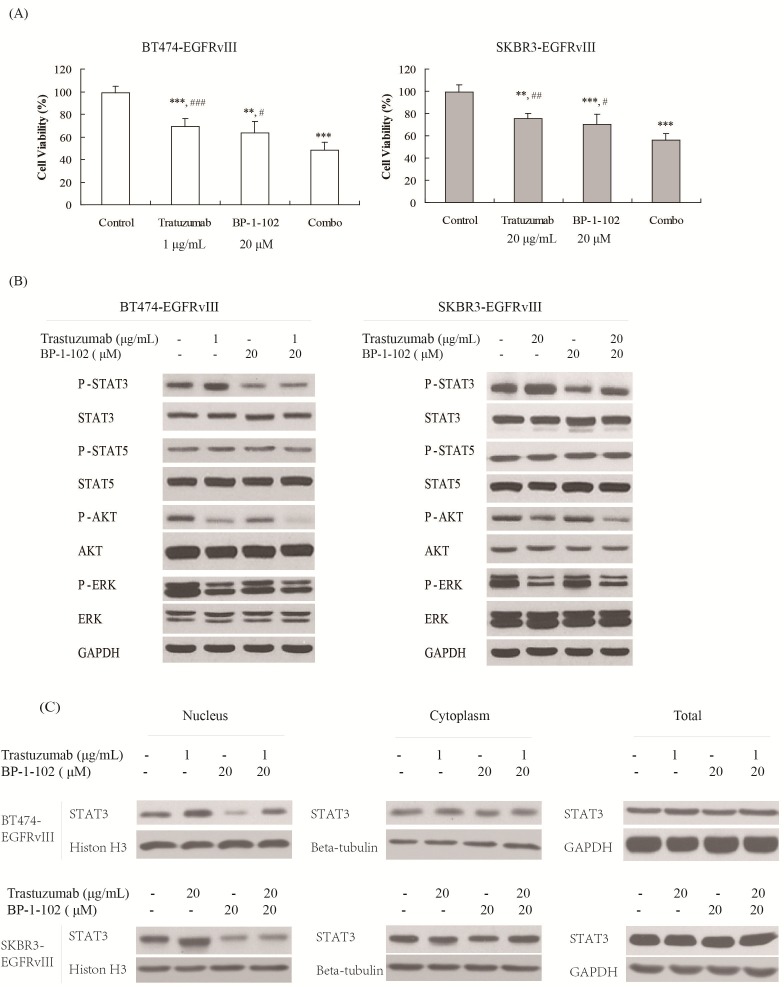
STAT3 inhibitor increased the antitumor efficacy of trastuzumab in EGFRvIII^+^HER2^+^ breast cancers **A.** The cytotoxicities effects of STAT3 inhibitor BP-1-102 in combination with trastuzumab on BT474-EGFRvIII and SKBR3-EGFRvIII cells. BT474-EGFRvIII cells were exposed to trastuzumab at a concentration of 1 μg/mL, BP-1-102 at a concentration of 20 μM or their combination for 48 hours. SKBR3-EGFRvIII cells were treated with trastuzumab at a concentration of 20 μg/mL or BP-1-102 at a concentration of 20 μM or the combination for 48 hours. Data are expressed as the cell viability in triplicate experiments (Bars, SD). *P < 0.05, **P < 0.01, ***P < 0.001 *versus* control group, # *P* < 0.05, ## *P* < 0.01, ### *P* < 0.001 *versus* combo group. **B.** Signaling events upon treatment with BP-1-102, trastuzumab or the combination in BT474-EGFRvIII and SKBR3-EGFRvIII cells. **C.** Immunoblots evaluating HER2 and STAT3 nucleus translocation in BT474-EGFRvIII and SKBR3-EGFRvIII cells after BP-1-102, trastuzumab or the combination treatment.

To determine the role of STAT3 in trastuzumab resistance in EGFRvIII^+^HER2^+^ breast cancers, the STAT3 inhibitor BP-1-102 was applied together with trastuzumab to treat breast cancer cells. The results illustrated that the combination of BP-1-102 and trastuzumab significantly enhanced the antitumor effect of trastuzumab on BT474-EGFRvIII and SKBR3-EGFRvIII (Figure 2A) cells. The similar results were also observed in the combination of STAT3 siRNA with trastuzumab (Figure S2). In the combination group, STAT3 phosphorylation was significantly inhibited, and nuclear translocation of STAT3 was obviously reduced (Figure 2B and 2C). Other EGFR downstream signals activated constitutively by EGFRvIII were not obviously affected (Figure 2B). These results further demonstrated that STAT3 activation contributed to trastuzumab resistance in EGFRvIII^+^HER2^+^ breast cancer cells.

### CH12 significantly suppressed the growth of EGFRvIII^+^HER2^+^ breast cancers *in vitro* and *in vivo*

**Figure 3:**
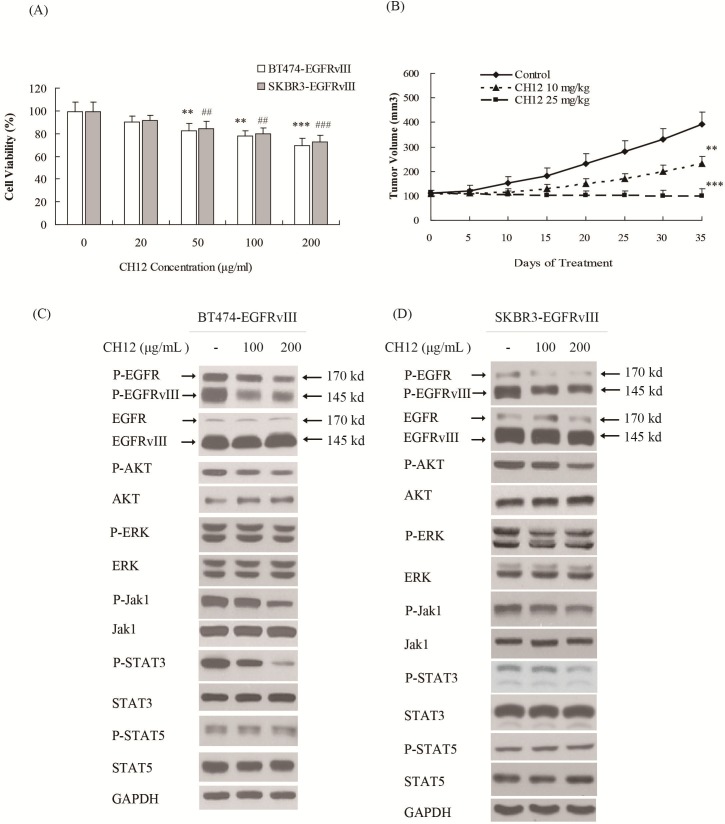
CH12 inhibited the growth of EGFRvIII^+^HER2^+^ breast cancer cells *in vitro* and *in vivo* **A.** The proliferation of BT474-EGFRvIII and SKBR3-EGFRvIII cells was inhibited by CH12 in a dose dependent manner. Cells were exposed to CH12 at concentrations of 20, 50, 100 and 200 μg/mL for 48 hours and were analyzed via a CCK-8 assay. Data are expressed as the cell viability in triplicate experiments (Bars, SD). Statistical signiﬁcance is indicated *versus* control *P < 0.05, **P < 0.01, ***P < 0.001 for BT474-EGFRvIII cells, and # *P* < 0.05, ## *P* < 0.01, ### *P* < 0.001 for SKBR3-EGFRvIII cells. **B.** Growth curve of BT474-EGFRvIII xenografts treated by vehicle, CH12 at a concentration of 10 mg/kg or 25 mg/kg, intraperitoneally, three times per week for 2 weeks. The data are expressed as mean tumor volumes ± SE. Statistical signiﬁcance is indicated *versus* vehicle *P < 0.05, **P < 0.01, ***P < 0.001. **C.**, **D.** The changes of relevant signaling pathway in BT474-EGFRvIII **C.** and SKBR3-EGFRvIII **D.** cells after CH12 treatment.

To determine whether an EGFRvIII-targeted strategy could be applied in breast cancer therapy, the anti-EGFRvIII monoclonal antibody CH12 was applied to treat EGFRvIII^+^HER2^+^ breast cancer cells. *In vitro* growth inhibition effect of CH12 was examined. The results shown in Figure 3A indicated that CH12 inhibited the growth of BT474-EGFRvIII and SKBR3-EGFRvIII cells in a dose-dependent manner. The antitumor effect of CH12 was further evaluated in established BT474-EGFRvIII tumor models. As shown in Figure 3B, CH12 significantly inhibited the growth of BT474-EGFRvIII xenografts with an inhibition rate of 41% at a dose of 10 mg/kg and 74% at a dose of 25 mg/kg on day 35 after the first administration (Figure 3B). Further study revealed that CH12 could obviously inhibit phosphorylation of EGFR, AKT, Jak1 and STAT3 (Figure 3C and 3D). Together, CH12 significantly suppressed the growth of EGFRvIII^+^HER2^+^ breast cancers *in vitro* and *in vivo via* inhibiting EGFR downstream signals.

### Combination of trastuzumab with CH12 synergistically inhibited the growth of EGFRvIII^+^HER2^+^ breast cancer cells *in vitro* and *in vivo*

**Figure 4:**
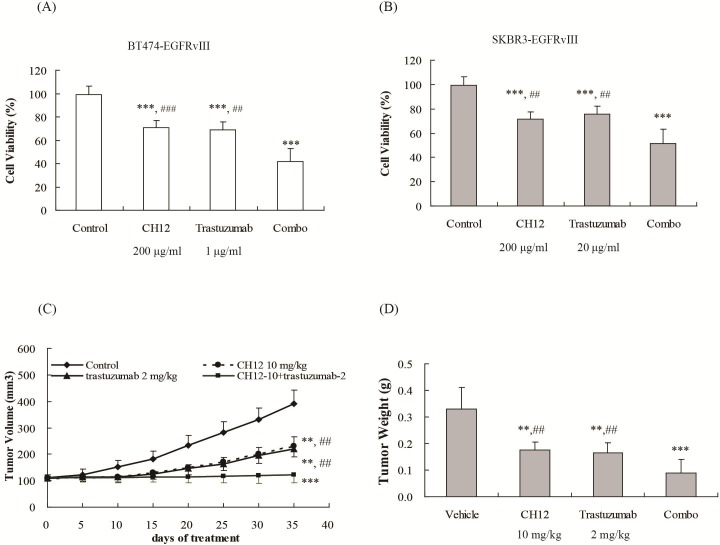
Combination of CH12 with trastuzumab suppressed growth of EGFRvIII^+^HER2^+^ breast cancers *in vitro* and *in vivo* **A.**, **B.** The efficacy of trastuzumab in combination with CH12 in BT474-EGFRvIII and SKBR3-EGFRvIII cells. **A.** BT474-EGFRvIII cells were exposed to CH12 at a concentration of 200 μg/mL, trastuzumab at a concentration of 1 μg/mL or the combination for 48 hours. **B.** SKBR3-EGFRvIII cells were treatment by CH12 at a concentration of 200 μg/mL, trastuzumab at a concentration of 20 μg/mL or the combination for 48 hours. Data are expressed as the cell viability in triplicate experiments (Bars, SD). **C.**, **D.** Growth curve **C.** and tumor weight **D.** of BT474-EGFRvIII xenografts upon intraperitoneally treatment with vehicle, trastuzumb at a concentration of 2 mg/kg weekly, CH12 at a concentration of 10 mg/kg three times a week or the combination for 2 weeks. Data are the mean tumor volumes ± SE. *P* < 0.05 was considered statistically significant. *P < 0.05, **P < 0.01, ***P < 0.001 *versus* control (veichle), # *P* < 0.05, ## *P* < 0.01, ### *P* < 0.001 *versus* combo group.

According to the efficacy of CH12 and the inhibition effect against ERK, AKT and Jak1/STAT3 pathway in EGFRvIII^+^HER2^+^ breast cancers, we wondered whether the combination of trastuzumab with CH12 could reverse trastuzumab resistance in EGFRvIII^+^HER2^+^ breast cancer. First, we determined the combination effect of trastuzumab and CH12 *in vitro*. The cell viability after treated by trastuzumab, CH12 and the combination were 69.6%, 68.6%, and 42.1% in BT474-EGFRvIII cells, and 72.2%, 75.6% and 50.5% in SKBR3-EGFRvIII cells. The results illustrated that the combination imposed a synergetic growth-inhibition effect on both BT474-EGFRvIII and SKBR3-EGFRvIII cells compared with either antibody alone (*P* < 0.01, CDI < 1 in both model, Figure 4A and 4B). To investigate the *in vivo* antitumor effect of the combination of trastuzumab with CH12, mice bearing BT474-EGFRvIII xenografts were treated with trastuzumab, CH12 or the combination. All animals tolerated the treatments well, without observable signs of toxicity, and had stable body weights during the study. The inhibitory ratios of trastuzumab, CH12 and the combination on day 35 after first administration were 41%, 43%, and 69%, respectively (Figure 4C), which suggested that tumor growth was synergistically inhibited by the combination treatment (*versus* trastuzumab or CH12 treatment alone, *P* < 0.01, CDI < 1). Tumor weight was also measured at the end of the study (Figure 4D), and the result was consistent with that of tumor volume. Taken together, these data indicated that the combination of trastuzumab and CH12 exhibited synergistic antitumor activity *in vitro* and *in vivo*.

### Combination of trastuzumab with CH12 suppressed the EGFR downstream pathway and STAT3 feedback activation

**Figure 5:**
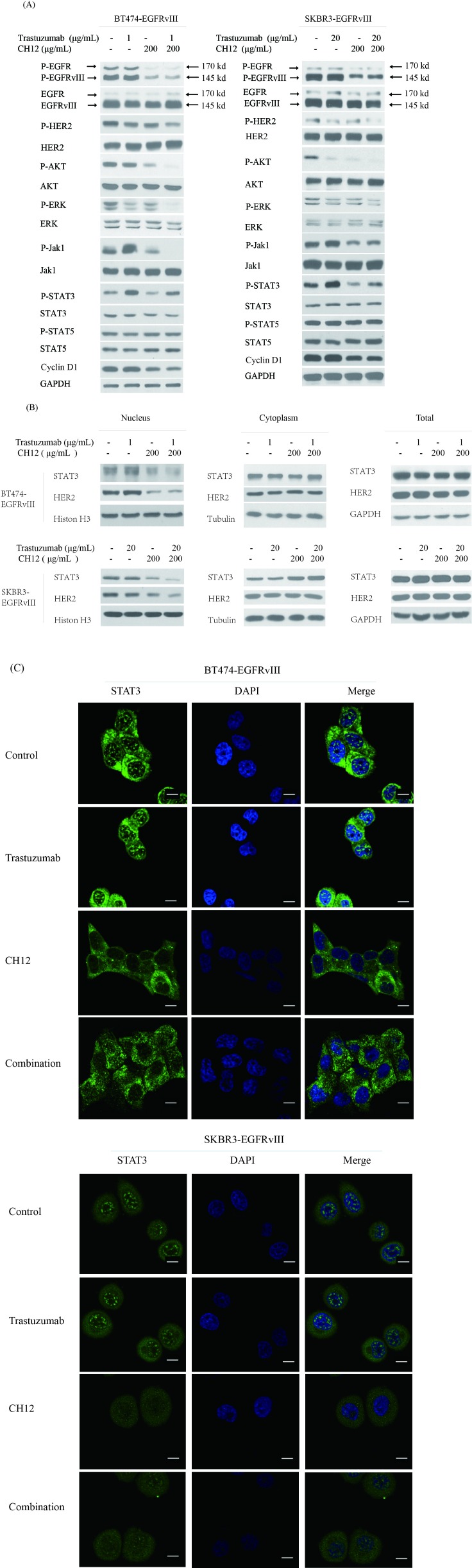
Combination of CH12 with trastuzumab suppressed the EGFR downstream pathway and STAT3 feedback activation **A.** Signaling events in BT474-EGFRvIII and SKBR3-EGFRvIII cells upon treatment with control, CH12, trastuzumab or the combination for 48 hours. **B.**, **C.** HER2 and STAT3 nucleus translocation evaluated by western blot **B.**, or STAT3 translocation evaluated by immunofluorescence **C.** after BT474-EGFRvIII and SKBR3-EGFRvIII cells treatment with control, CH12, trastuzumab or the combination for 48 hours. The scale bars equal to 20 μm **C.**.

To gain further insight into the molecular events occurring in combination-treated cells, certain key signaling molecules were examined *via* Western blot analysis. The phosphorylation level of AKT and ERK in the combination treatment cells was lower than those in the monotherapy groups, and the phosphorylation of Jak1 and STAT3 was also significantly inhibited compared with the trastuzumab group (Figure 5A and 5B). Similar changes of these molecules were also observed in the BT474-EGFRvIII xenograft models (Figure S3).

STAT3 need to dimerize to transmit signals to the nucleus and function as a transcription factor [32]. It was reported that the STAT3/HER2/HER3 complex could translocate to the nucleus and promote cyclinD1 translation [33], which is a vital cell cycle regulator for cell cycle progression in phase G1. Therefore, we examined the nuclear translocation of HER2 and STAT3. The results showed that combination treatment inhibited their nuclear translocation (Figure 5B and 5C) and reduced the expression of cyclin D1 (Figure 5A and 5B). Together, the combination inhibited the AKT, ERK and Jak1/STAT3 pathways more efficiently when compared with trastuzumab monotherapy. The combination treatment also strongly inhibited STAT3 and HER2 nuclear translocation and reduced the cyclin D1 expression.

### Combination of trastuzumab with CH12 potently reduced proliferation and angiogenesis, and induced apoptosis in EGFRvIII^+^HER2^+^ breast cancers

**Figure 6:**
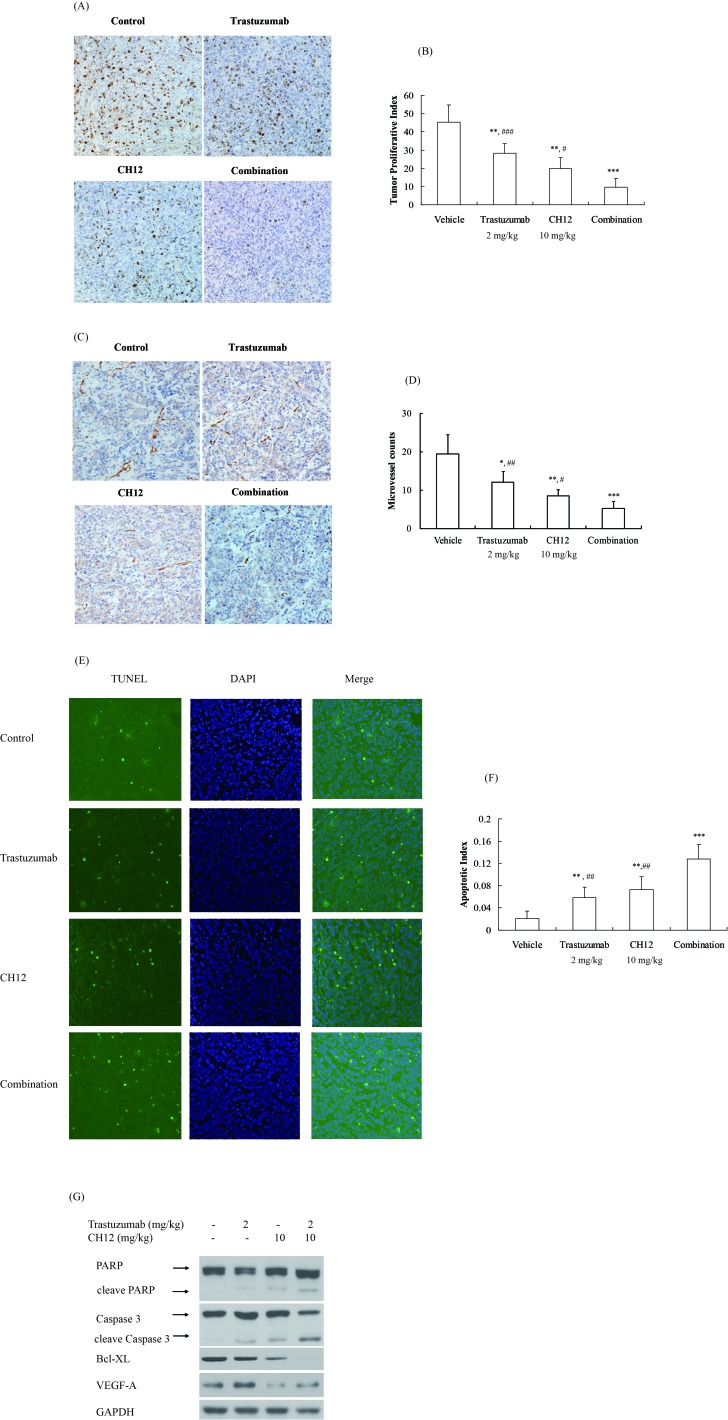
Combination of CH12 with trastuzumab reduced proliferation and angiogenesis and induced apoptosis in the tumor xenografts **A.** Trastuzumab combination with CH12 treatment led to less growth compared with other treatments in BT474-EGFRvIII xenograft. Tumor sections were stained for Ki-67. The cell proliferative index was assessed as the percentage of total cells that were Ki-67 positive from six randomly selected high power fields (×200) in xenografts from six mice of each group. **B.** The quantitative analysis of Ki-67 staining. **C.** Trastuzumab combination with CH12 treatment led to less vascularization compared with control in BT474-EGFRvIII xenograft. Tumor sections were immunostained with anti-CD34 antibody. MVD values were analyzed by measuring the number of stained microvessels from six randomly selected fields (×200) in xenografts from six mice of each group. **D.** The quantitative analysis of CD34 staining. **E.** Combination treatment led to an increase in apoptosis compared with control in BT474-EGFRvIII xenografts. Apoptotic cells were detected using the TUNEL assay. The apoptotic index was assessed by the ratio of TUNEL-positive cells : total number of cells from six randomly selected high power fields (×200) in xenografts from six mice of each group. **F.** The quantitative analysis of TUNEL assay. **G.** Signaling events related to apoptosis and angiogenesis upon treatment with CH12, trastuzumab or the combination in BT474-EGFRvIII xenograft. Data are the mean ± SD. *P < 0.05, **P < 0.01,***P < 0.001 *versus* veichle, # *P* < 0.05, ## *P* < 0.01, ### *P* < 0.001 *versus* combo group.

To further elucidate the causes underlying the *in vivo* synergistic activity of trastuzumab and CH12, the proliferative index, tumor microvessel density as well as the apoptotic index was evaluated. The proliferative index was significantly lower in the combination-treatment group than the vehicle group as well as the monotherapy groups (*P* < 0.05 monotherapy *versus* combination; Figure 6A and 6B).

The number of the CD34-positive microvessels in the combination-treatment group was also significantly less than that in the monotherapy groups (*P* < 0.05 monotherapy *versus* combination; Figure 6C and 6D). VEGF-A expression in CH12 and combination group abated significantly when compared with control and trastuzumab group (*P* < 0.05, Figure 6G).

TUNEL staining demonstrated a significant increase in the number of apoptotic cells in the combination-treatment group compared with the monotherapy groups (*P* < 0.01, Figure 6E and 6F). Consistently, cleaved caspase 3 and cleaved PARP increased obviously while the expression of Bcl-XL, an anti-apoptosis protein reduced significantly (Figure 6G). Together, these data showed that trastuzumab and CH12 combination therapy had a stronger effect on reducing tumor proliferation and angiogenesis and inducing tumor cell apoptosis than monotherapy *in vivo*, leading to synergistic tumor inhibition.

## DISCUSSION

Although EGFRvIII has been demonstrated to co-express with HER2 in breast cancer, its contribution to the sensitivities of breast cancer against trastuzumab has not been determined. Our study indicated that EGFRvIII expression could decrease the sensitivity of breast cancer cells against trastuzumab *via* activating the ERK, AKT and Jak1/STAT3 pathways (Figure 1), which suggested that EGFRvIII inhibitor might reduce trastuzumab resistance.

Intriguingly, we observed that the Jak1/STAT3 pathway was significantly activated in trastuzumab-treated EGFRvIII^+^HER2^+^ breast cancer cells. STAT3 is constitutively activated in many types of human cancers, including breast cancer, and plays crucial roles in regulating tumor cell proliferation, survival, invasion, and angiogenesis [32, 34]. STAT3 activation has also been reported as a vital cause contributing to trastuzumab resistance in HER2^+^ breast and gastric cancer [35]. In approximately 50-60% of the primary breast tumors, STAT3 has been reported constitutively activated through phosphorylation by cytoplasmic non-receptor tyrosine kinases [36, 37]. Accumulating evidence has demonstrated that STAT3 activation is also associated with drug resistance as a considerable feedback loop and limits the efficacy of agents [38–40], such as various RTK inhibitors [41]. Furthermore, it was reported that STAT3/HER2/HER3 complex translocates to the nucleus and promotes cyclinD1 translation, which is a major driver of in trastuzumab resistance [32]. In this study, we revealed that STAT3 inhibition significantly increased the antitumor effect of trastuzumab on EGFRvIII^+^HER2^+^ breast cancers (Figure 2), implicating that STAT3 inhibitors might be used to increase the antitumor effect of trastuzumab on EGFRvIII^+^HER2^+^ breast cancers..

Our previous data demonstrated that mab CH12 could selectively bind to EGFRvIII and significantly suppress the growth of EGFRvIII-positive tumor xenografts [26]. Here we demonstrated that CH12 had a significant growth-suppression effect on EGFRvIII^+^HER2^+^ breast cancers *in vitro* and *in vivo* (Figure 3A and 3B) at least partially through inhibiting the phosphorylation of EGFR, AKT and STAT3 (Figure 3C and 3D). Thus, it seems rational to combine CH12 and trastuzumab, which have opposite role on STAT3 phosphorylation.

As we expected, CH12 in combination with trastuzumab exhibited synergistic antitumor activity in EGFRvIII^+^HER2^+^ breast cancers *in vitro* and *in vivo* (Figure 4). The combination inhibited the ERK or AKT pathway more efficiently and abrogated the feedback activation of STAT3 caused by trastuzumab treatment. Additionally, the combination lessened the nuclear translocation of STAT3 and HER2 and reduced cyclin D1 expression, leading to increased efficacy (Figure 5). Furthermore, compared with the monotherapy, the combination treatment significantly suppressed tumor proliferation and microvessel density while significantly induced apoptosis (Figure 6). It has been reported that STAT3 phosphorylation promotes angiogenesis various cancer types including breast cancer and the expression of angiogenic factor VEGF-A plays a vital role in STAT3-induced angiogenesis [42–44]. In this study, VEGF-A expression and the microvessel density in the combination group reduced significantly than trastuzumab monotherapy group (Figure 6C, 6D and 6G), indicating that anti-angiogenesis played important role in the antitumor activities of the combination treatment.

It is well known that the toxic side effects on normal tissue present huge obstacles in cancer treatment and lead to dose reductions, treatment delays, and even the discontinuation of therapy. In this study, we demonstrated that STAT3 inhibitor could increase the antitumor activities of trastuzumab. However, STAT3 inhibitor could not distinguish the STAT3 in normal cells and cancer cells and might cause severe side effects. In contrast, CH12 targets a cryptic epitope of EGFR or EGFRvIII, which only exposes in tumor cells and not in normal tissue, leading to a good safety profile. Clinically, mab 806, which targets a similar epitope as CH12, was well tolerated at all dose levels with generally predictable and manageable minor toxicities being observed [45]. Thus, the combination of trastuzumab with CH12 might show a larger safety window when compared with the combination of trastuzumab with JAK1/STAT3 inhibitors.

In summary, EGFRvIII expression increased the *de novo* resistance of breast cancer cells against trastuzumab, and the feedback activation of STAT3 caused by trastuzumab also contributed to acquired resistance in EGFRvIII^+^HER2^+^ breast cancers. Moreover, the combination of trastuzumab and CH12 could synergistically inhibit the growth of EGFRvIII^+^HER2^+^ breast cancer *via* attenuating phosphorylation ERK and AKT more effectively and reversing STAT3 feedback activation. Taken together, we propose that the combination of Trastuzumab and CH12 might be a novel treatment strategy for the patients with EGFRvIII^+^HER2^+^ breast cancer.

## MATERIALS AND METHODS

### Cell culture

The human breast cancer cells BT474 (HER2/ER-positive) and SKBR3 (HER2-positive/ER-negative) were obtained from American Type Culture Collection. BT474 and SKBR3 with exogenous EGFRvIII overexpression were established according to previously reported methods [27]. All breast cancer cells were cultured in 1640 medium (Gibco, USA) supplemented with 10% fetal bovine serum (Serana, Australia) and maintained at 37°C in a humidified atmosphere of 5% CO_2_.

### Reagents

Trastuzumab was purchased from Genentech, USA and dissolved in PBS buffer at a concentration of 22 mg/mL. The chimeric mAb CH12 (IgG1) was produced in dihydrofolate reductase-deficient CHO DG44 cells as described previously at 20 mg/mL [26].

### FACS analysis

1×10^6^ cells were collected by centrifugation and incubated with 20 μg/mL primary antibody, in phosphate-buffered saline containing 1% newborn calf serum for 45 min at 4°C. After being washed with cold phosphate-buffered saline, cells were incubated for an additional 45 min at 4°C with an FITC-conjugated goat anti-human antibody (Kang-Chen Bio-tech, Shanghai, China) in the dark. For each sample, at least 10,000 cells were analyzed by FACS cytometry (Beckman Coulter Epics Altra, Miami, FL).

### RNA interference assay

STAT3 siRNA (5′-AGUCAGGUUGCUGGUCAAA-3′) and negative control siRNA (siN05815122147) were purchased from Ribobio, Guangzhou, China [28]. The cells were plated at 50% confluence, transfected with 50 nM siRNA overnight in Opti-MEM containing Lipofectamine 2000 (Invitrogen), and incubated for various amounts of time.

### *In vitro* cell proliferation assay

The cells were seeded in 96-well plates at a density of 5000 cells per well. After 24 hours, the cell media was replaced with media containing trastuzumab or CH12 at different concentrations or PBS (vehicle), using five wells per concentration. After 48 hours, cell proliferation was measured using a CCK-8 kit (Dojindo Laboratories, Rockville, MD). CCK-8 solution (10 μl) was added to 90 μl of culture media and incubated for 1 h at 37°C in a humidified atmosphere of 5% CO_2_. The optical density was measured at 450 nm in a microplate reader. The experiment was performed in triplicate at different times.

### Western blotting analysis

Protein lysates were obtained from BT474-EGFRvIII and SKBR3-EGFRvIII cells using a lysis buffer (Prod# 78501, Thermo, USA) with a protease inhibitor cocktail. Cell lysates were collected after centrifugation at 12,000 *g* for 10 minutes at 4°C. The tumor tissues were surgically excised and frozen in liquid nitrogen and then homogenized in tumor lysis buffer (Prod# 78510, Thermo, USA); after centrifugation at 12,000 *g* for 10 minutes at 4°C, the lysates were collected. The protein was quantified using a BCA Kit (Prod# 23225, Thermo, USA), separated on SDS-PAGE gels at 8%-14% polyacrylamide according to protein weight and blotted onto a PVDF nitrocellulose membrane (Bio-Rad Laboratories, USA). The membrane was blocked in 5% milk in PBST for 1 hour and then probed with primary antibodies overnight at 4°C. The following primary antibodies were used: the phosphor-HER2, HER2, phosphor-EGFR, EGFR, phosphor-ERK, ERK, Bcl-xL, and p27 antibodies purchased from Santa Cruz Biotechnology, and the cleaved caspase3, caspase3, PARP, phosphor-Akt (Ser473), Akt, Jak1, phosphor-Jak1, STAT5, phosphor-STAT5, STAT3 and phosphor-STAT3 (Tyr705) antibodies obtained from Cell Signaling Technology. After the membranes were washed in PBST and incubated with the appropriate secondary antibodies for 1 h at room temperature, washed three times in PBST and then visualized with enhanced chemiluminescence reagent, following the manufacturer's instructions (Prod# 34080, Thermo, USA).

### Tumor xenograft studies

All mouse experiments were performed in accordance with approved protocols from Shanghai Medical Experimental Animal Care Commission. BT474-EGFRvIII cells (1×10^7) in 100 μl of 1640-Matrigel mixture (1:1 ratio) were injected subcutaneously in the right lateral flank of 6-week-old nude mice. When the tumor volumes reached an average of approximately 100 mm^3^, we treated the mice with vehicle control (three times per week), trastuzumab (once per week), CH12 (three times per week) or trastuzumab-CH12 combination intraperitoneally. Tumor volumes were measured every five days in two dimensions with vernier calipers. The tumor volumes were calculated using the following formula: length × width^2^ × 0.5. Two weeks after the final treatment, the mice were sacrificed and the tumors were surgically excised and weighed. Tumor tissues from the *in vivo* experiments were collected for Western blot analysis and immunohistochemical studies.

### Immunohistochemical (IHC) analysis

To assess angiogenesis and cell proliferation in tumors, formalin-fixed paraffin-embedded tumor tissues were immunostained using monoclonal antibodies anti-CD34 (Abcam, Cambridge, UK) and anti-Ki-67 (Santa Cruz Biotechnology, USA). After deparaffinization and rehydration, the tissue sections were incubated with 3% hydrogen peroxide in methanol to quench endogenous peroxidase. The sections were blocked for 30 minutes with 1% BSA and incubated with the primary antibodies at 4°C overnight. As negative controls, staining was performed in the absence of the primary antibodies. The sections were then washed with PBS and incubated with HRP-conjugated secondary antibodies for one hour. The products were then visualized using a diaminobenzidine staining kit (TIANGEN Biotech, Beijing, China) and counterstained in hematoxylin.

As a measure of proliferation, the Ki-67 labeling index was determined as the ratio of (labeled nuclei) / (total nuclei) in high power fields (× 200). Approximately 2000 nuclei were counted in each case by systematic random sampling.

Microvessel density (MVD) was determined by measuring the number of stained microvessels in each section from six mice of each group as described [29]. The mean microvessel count of the six most vascular areas was taken as the MVD, which was expressed as the absolute number of microvessels per 0.74 mm^2^ (× 200 field).

### TUNEL assay

The TUNEL assay was performed according to kit instructions (Qia39, Merck, USA). The tumor tissues sections were deparaffinized, rehydrated and incubated with proteinase K (20 μg/mL) for 20 minutes at 37°C. After several washes with TBS, the specimen was covered in 1× equilibration buffer for 30 minutes, and the incubated with a mixture of 57.0 μl of Fluorescein-FragEL™ TdT Labeling Reaction Mix and 3 μl of TdT Enzyme for 1.5 hours at 37°C in the dark. Then, the slides were rinsed in TBS three times. A glass coverslips were mounted using Fluorescein-FragEL™ Mounting Media and slides were visualized under a fluorescence microscope (OLYMPUS IX71, Japan). TUNEL-positive cells were counted at ×400 magnification. The apoptotic index was calculated as a ratio of (apoptotic cell number) / (total cell number) in each field.

### Evaluation of the combination effect

The coefficient of drug interaction (CDI) was used to evaluate the combination effect. CDI is calculated in *in vitro* CCK-8 assay as follows: CDI = AB/(A×B). AB is the cell viability of combination group and A or B is the cell viability of antibody monotherapy group. In *in vivo* tumor xenograft model, end point tumor sizes were analyzed for combination effect using the formula CDI = (AB/C) / (A/C × B/C), where C is the tumor volume of the vehicle group, A or B is the tumor volume of antibody monotherapy group, and AB is the tumor volume of the combination group. CDI value < 1, =1 or >1 indicates that the combination is synergistic, additive or antagonistic, respectively [30, 31].

### Statistical analysis

All data are presented as the mean ± SE or mean ± SD. Statistical significance was determined by paired or unpaired Student *t* test in cases of standardized expression data. One-way ANOVA was performed for multiple group comparisons and comparisons between two groups were conducted using the least signiﬁcant difference method. *P* < 0.05 was considered significant.

## SUPPLEMENTARY MATERIAL FIGURES


