# Supplementary material for: Synergistic antitumor efficacy against the EGFRvIII+HER2+ breast cancers by combining trastuzumab with anti-EGFRvIII antibody CH12
Source: Oncotarget. 2015 Oct 14;6(36):38840–53. doi: 10.18632/oncotarget.6111 (PMC4770741; doi:10.18632/oncotarget.6111)
Supplement: Supplementary file 1 [file oncotarget-06-38840-s001.pdf]

Synergistic antitumor efficacy against the EGFRvIII^{+}HER2^{+} breast cancers by combining trastuzumab with anti-EGFRvIII antibody CH12

Supplementary Material

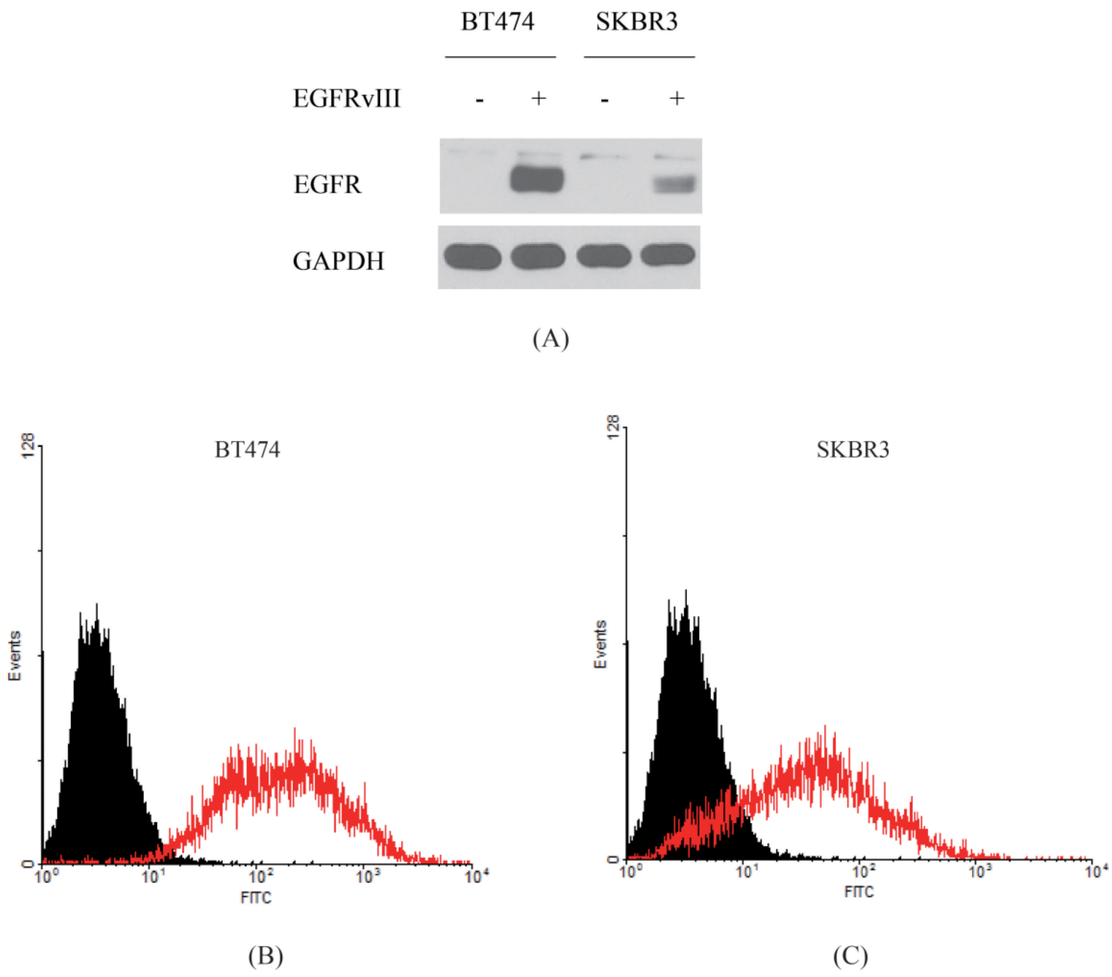

Figure S1 Construction of breast cancer cells with EGFRvIII overexpression.
(A) Western blot assay to analyze EGFRvIII expressin in BT474 and SKBR3 cell lines after infected with viral vectors encoding EGFRvIII .
(B, C) The binding capacity of CH12 to EGFRvIII^{+} BT474 (B) and EGFRvIII^{+} SKBR3 (C) cell lines was determined by FACS assay. Blank: parent cells, red: EGFRvIII-overexpressing cells.

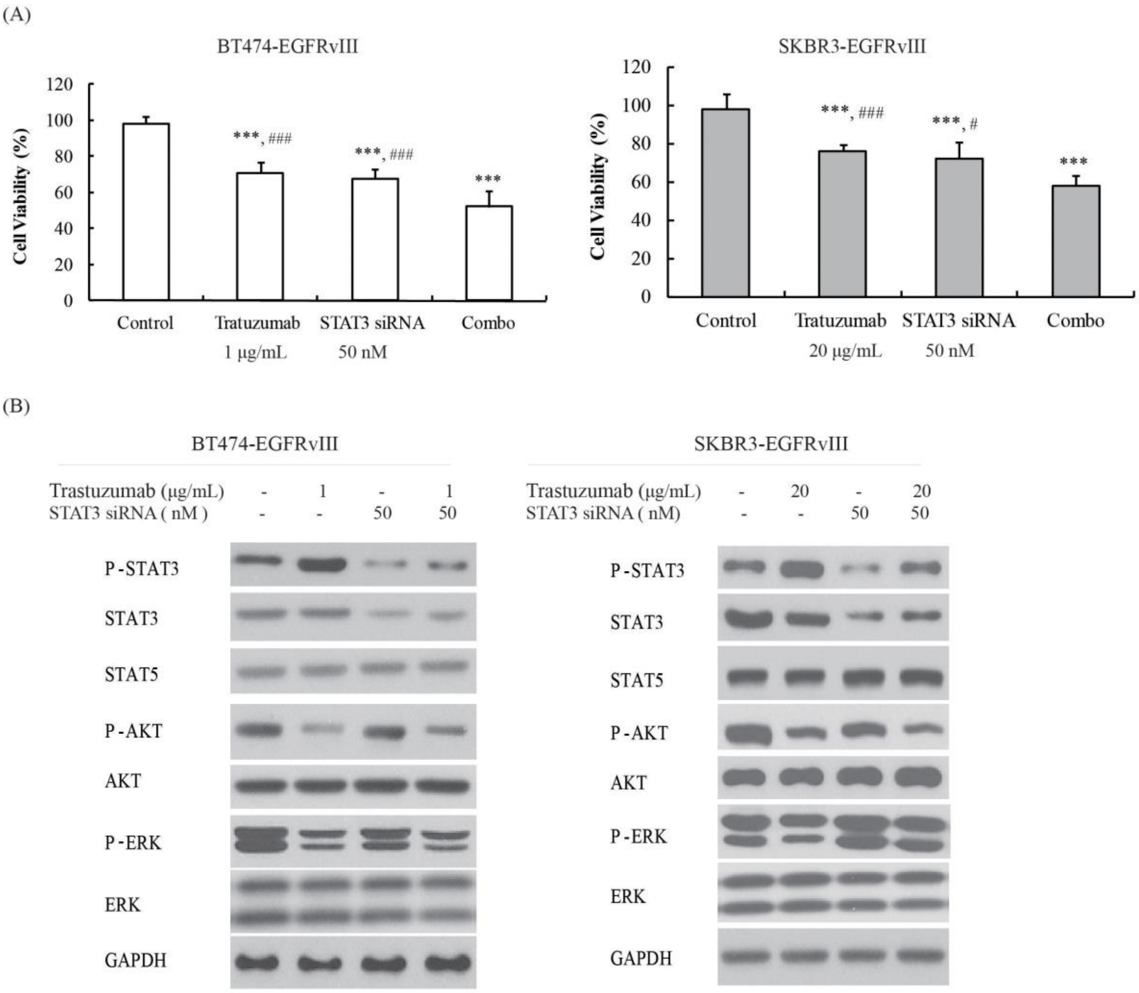

Figure S2 STAT3 siRNA increased the antitumor efficacy of trastuzumab in EGFRvIII^{+}HER2^{+} breast cancers.

(A) Cell growth effect of STAT3 siRNA in combination with trastuzumab in BT474-EGFRvIII and SKBR3-EGFRvIII cells. BT474-EGFRvIII cells were exposed to trastuzumab at a concentration of 1 μg/mL or STAT3 siRNA at a concentration of 50 nM or the combination for 48 hours. SKBR3-EGFRvIII cells were treatment by trastuzumab at a concentration of 20 μg/mL or STAT3 siRNA at a concentration of 50 nM or the combination for 48 hours. Data are expressed as the cell viability in triplicate experiments (Bars, SD). Statistical significance is indicated * P <0.05, ** P <0.01, *** P <0.001 versus control group, # P <0.05, ## P <0.01, ### P <0.001 versus combo group..

(B) Signaling events upon treatment with STAT3 siRNA, trastuzumab or the combination in BT474-EGFRvIII and SKBR3-EGFRvIII cells.

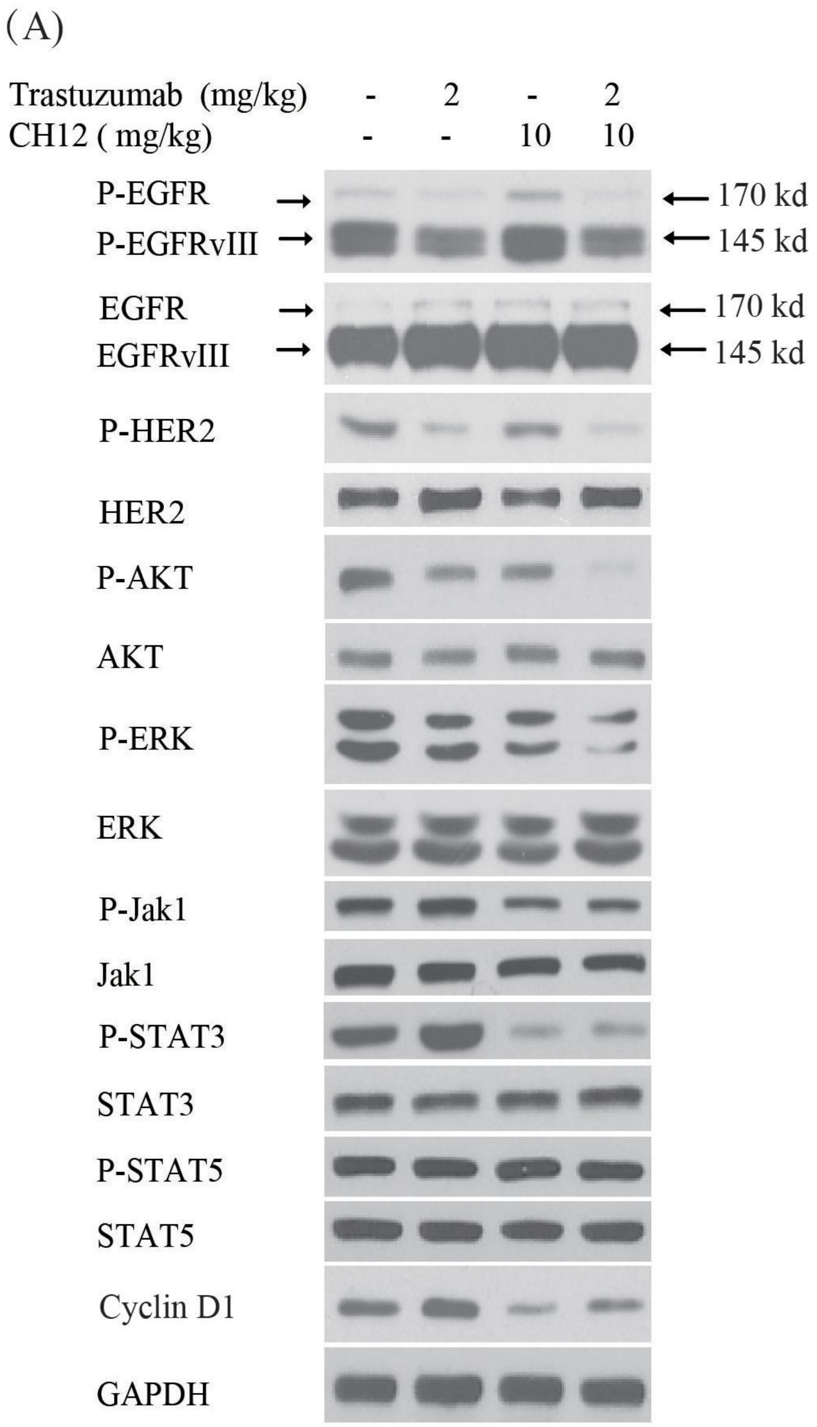

Figure S3 Combination of CH12 with trastuzumab inhibited the EGFR downstream pathway and STAT3 activation *in vivo*.

(A) Key molecules of EGFR pathway in BT474-EGFRvIII xenograft upon intraperitoneally administration of vehicle, trastuzumb at a concentration of 2 mg/kg weekly, CH12 at a concentration of 10 mg/kg for 3 times a week or their combination.
